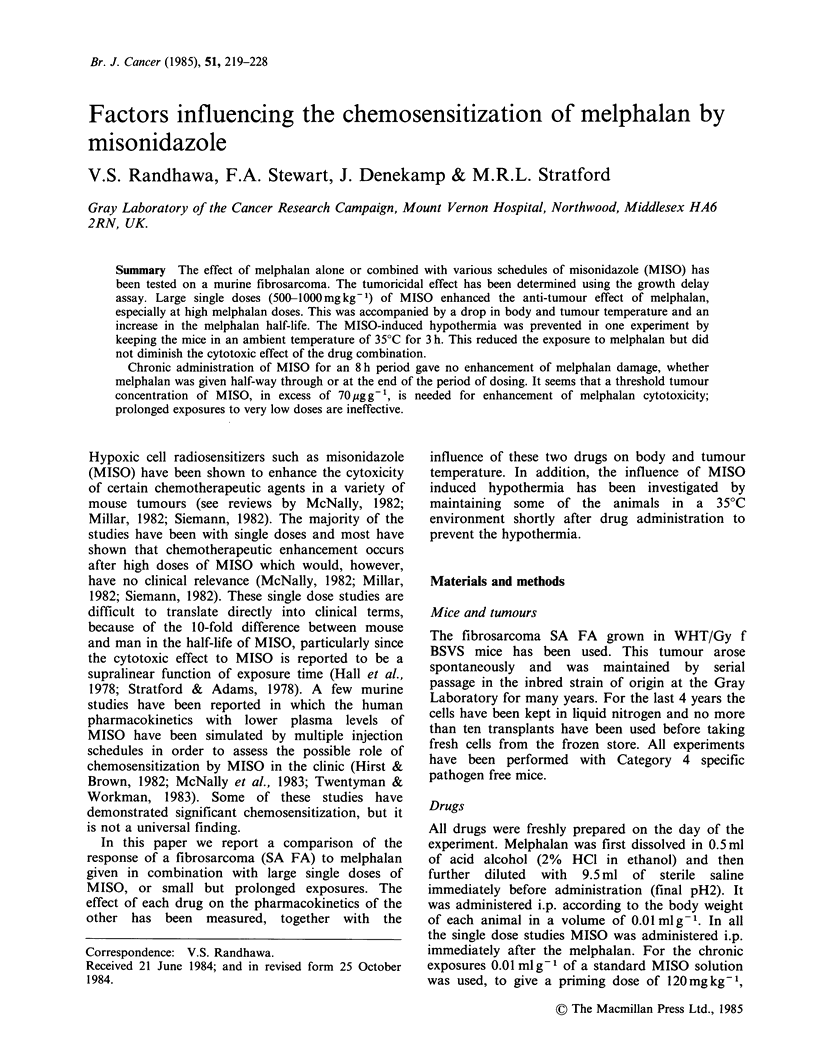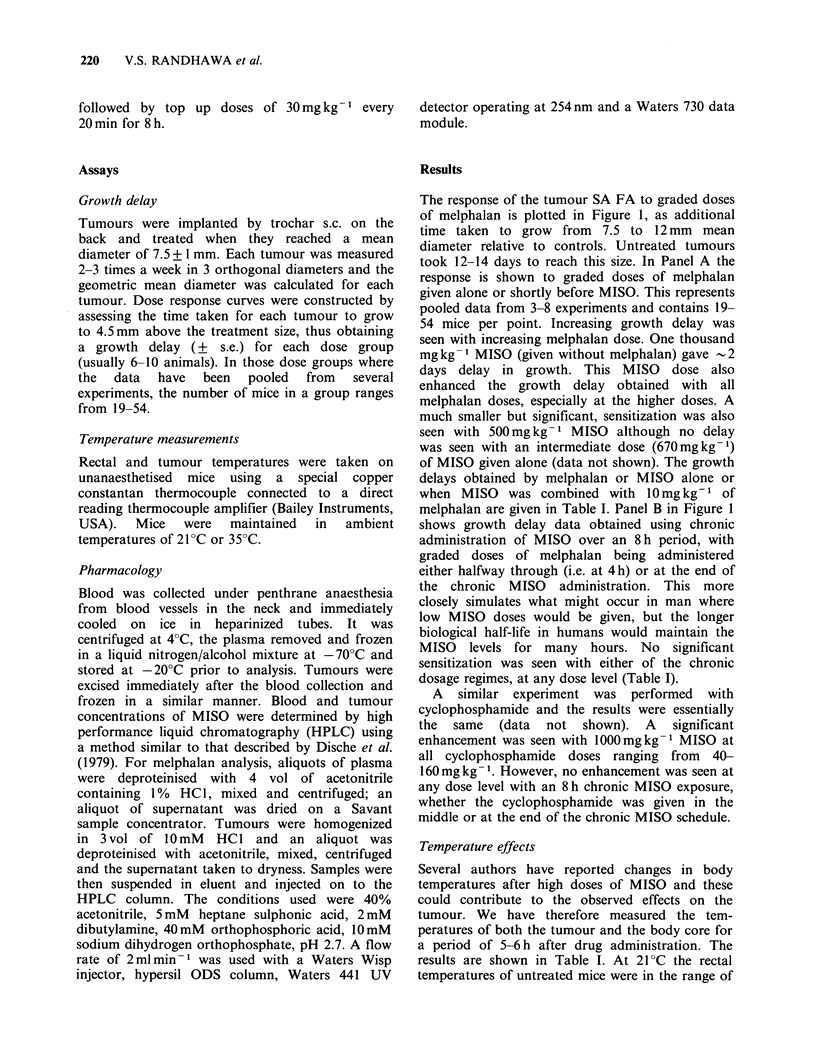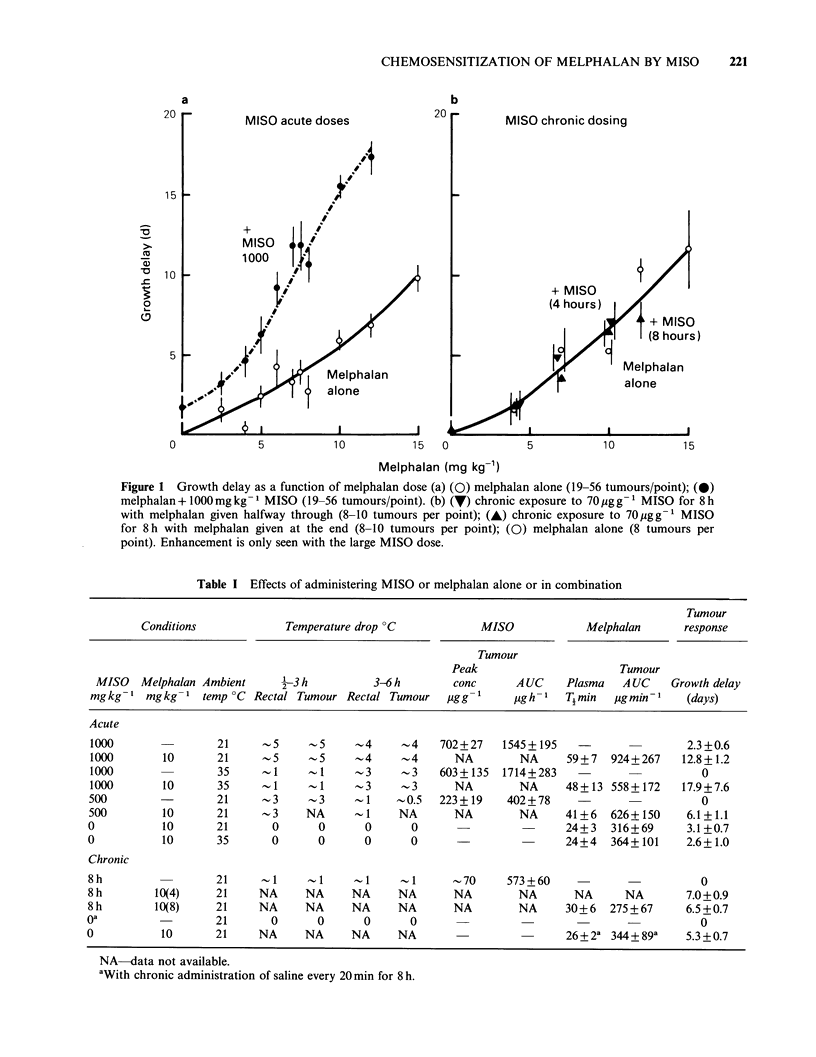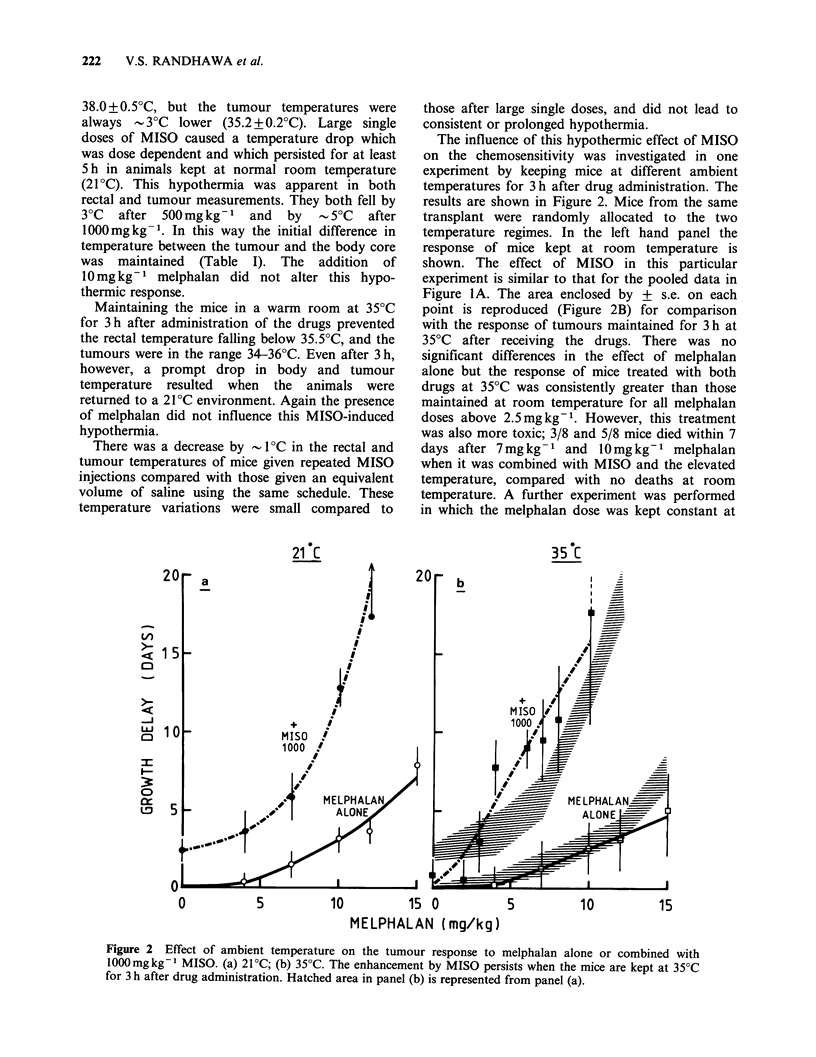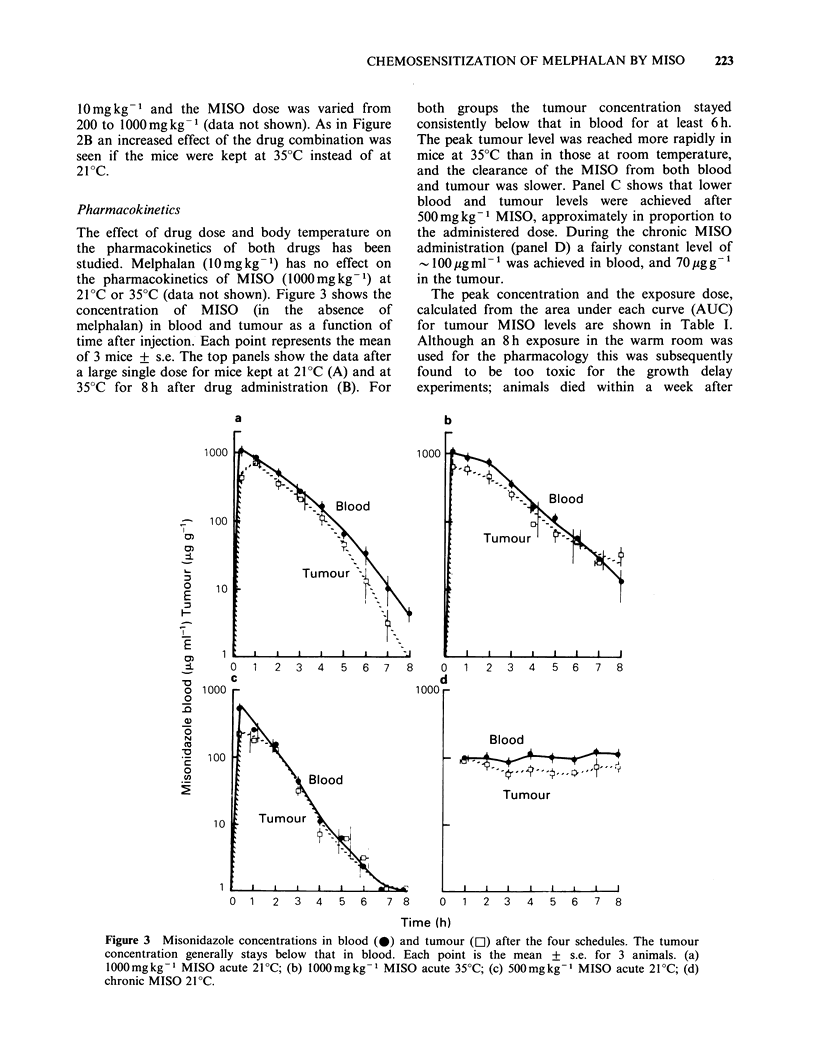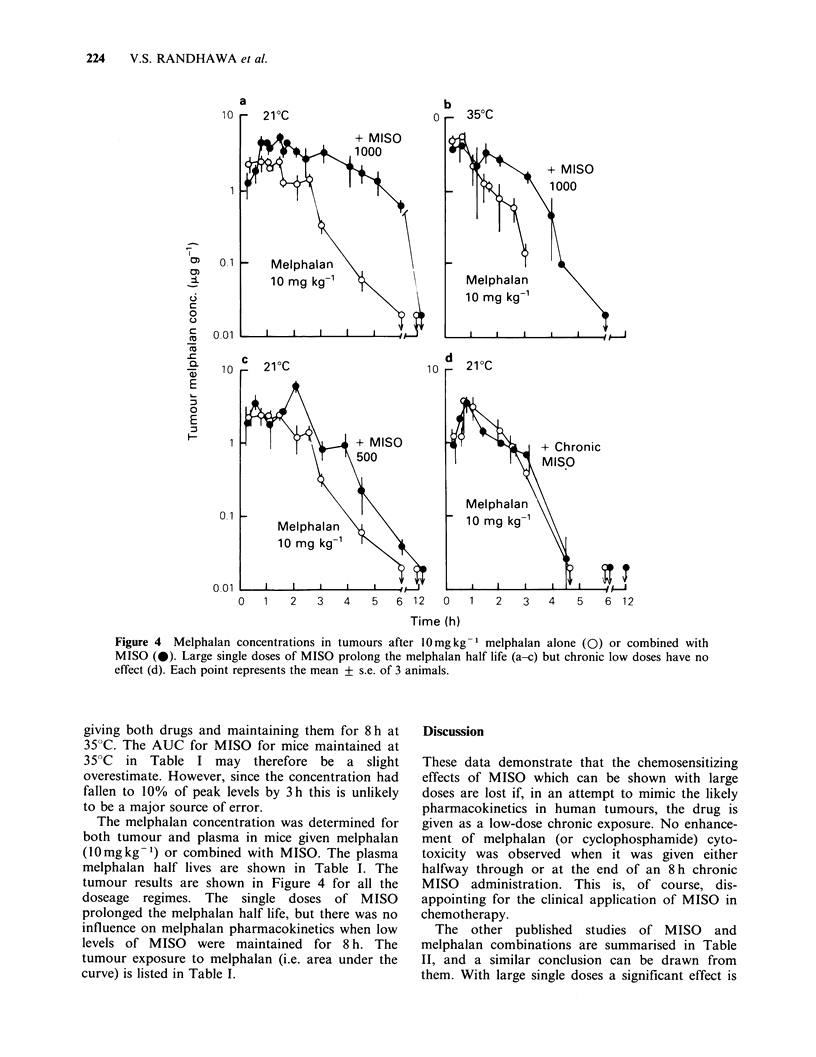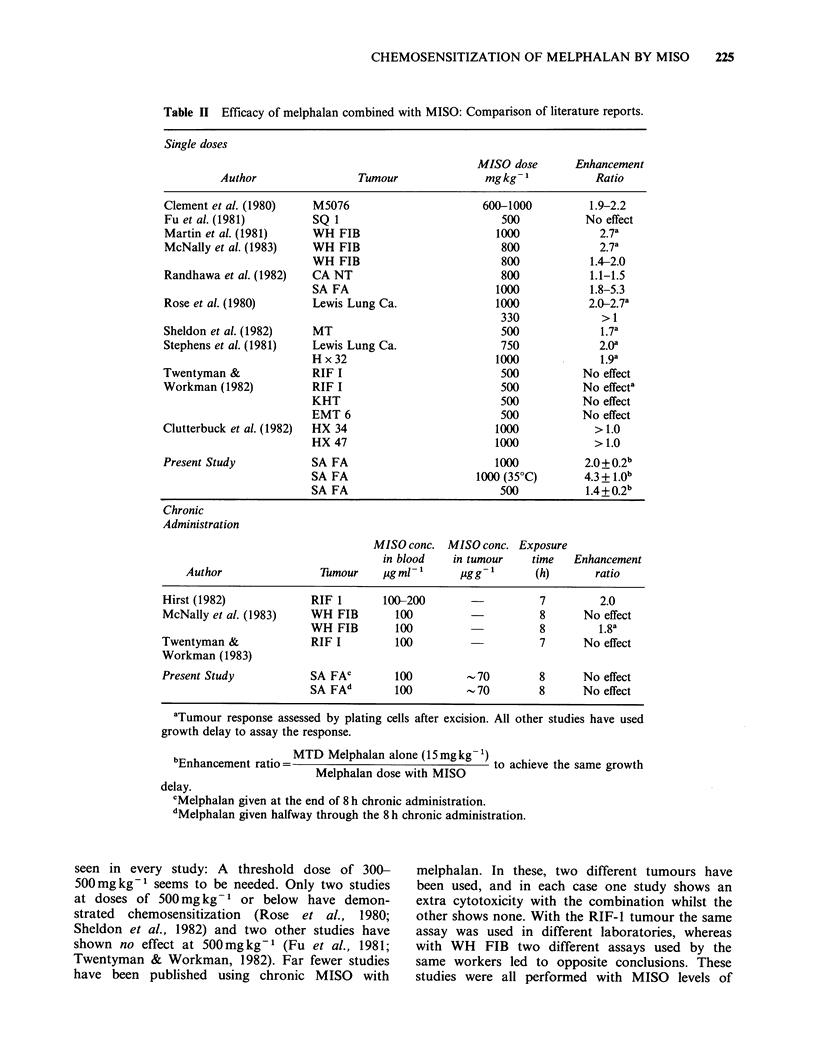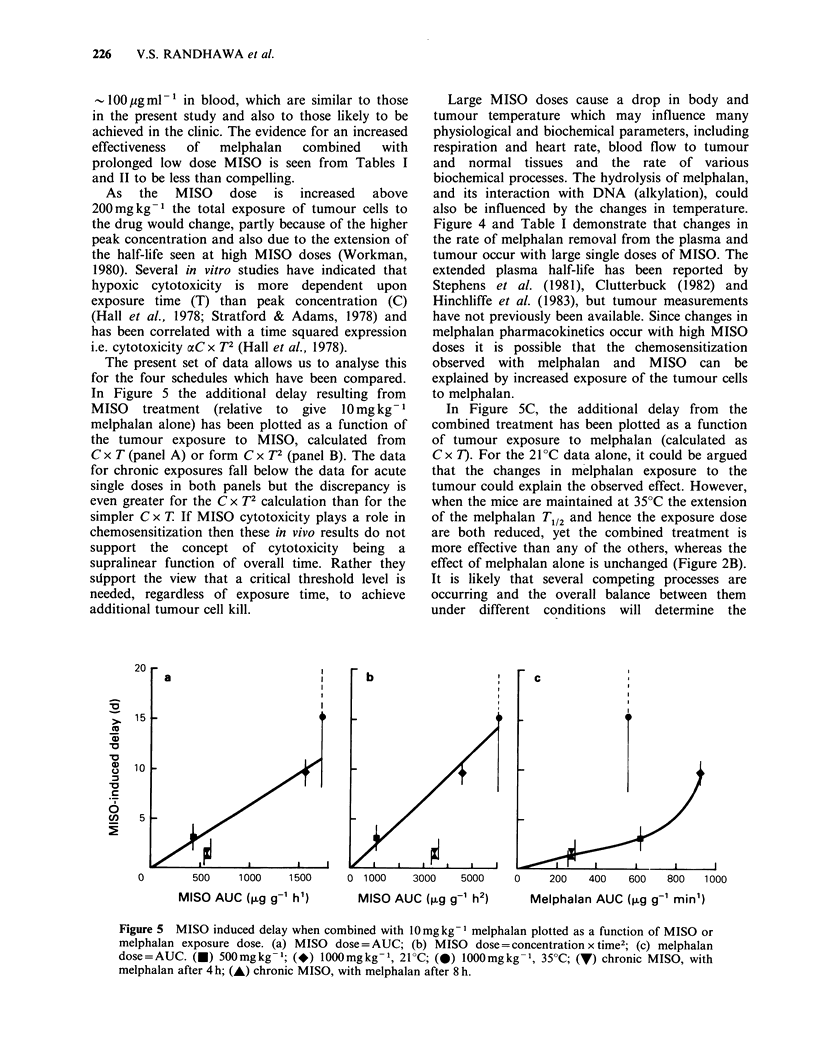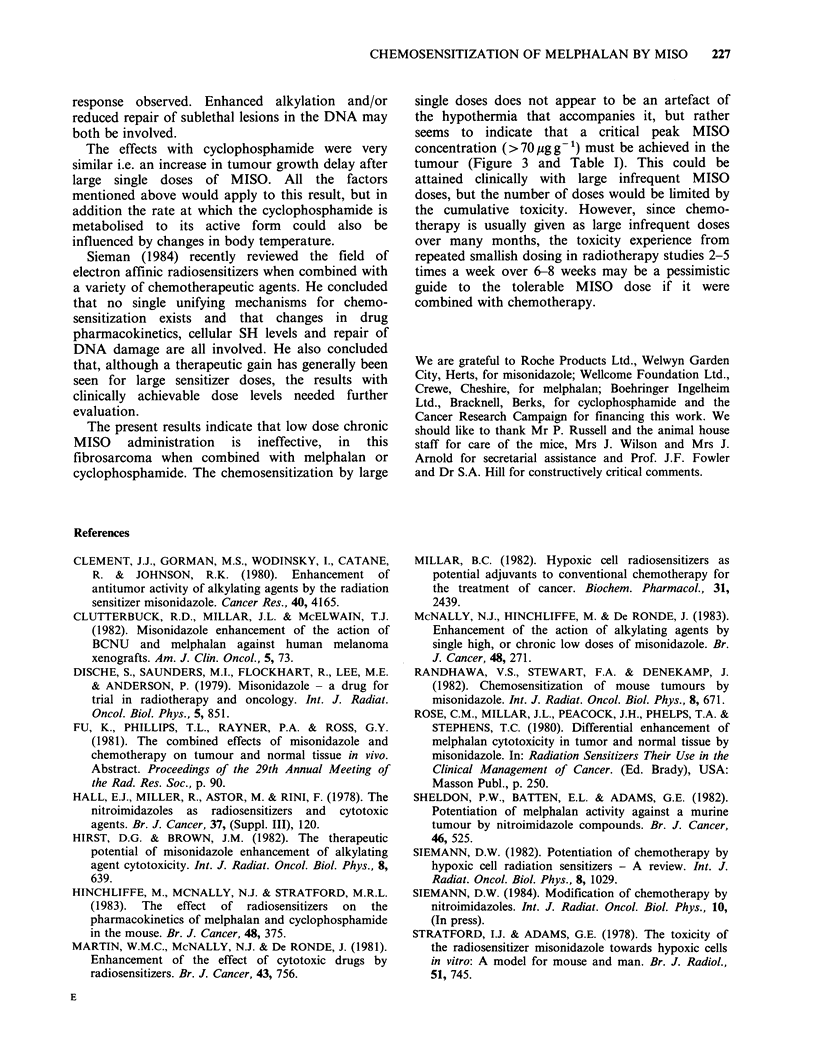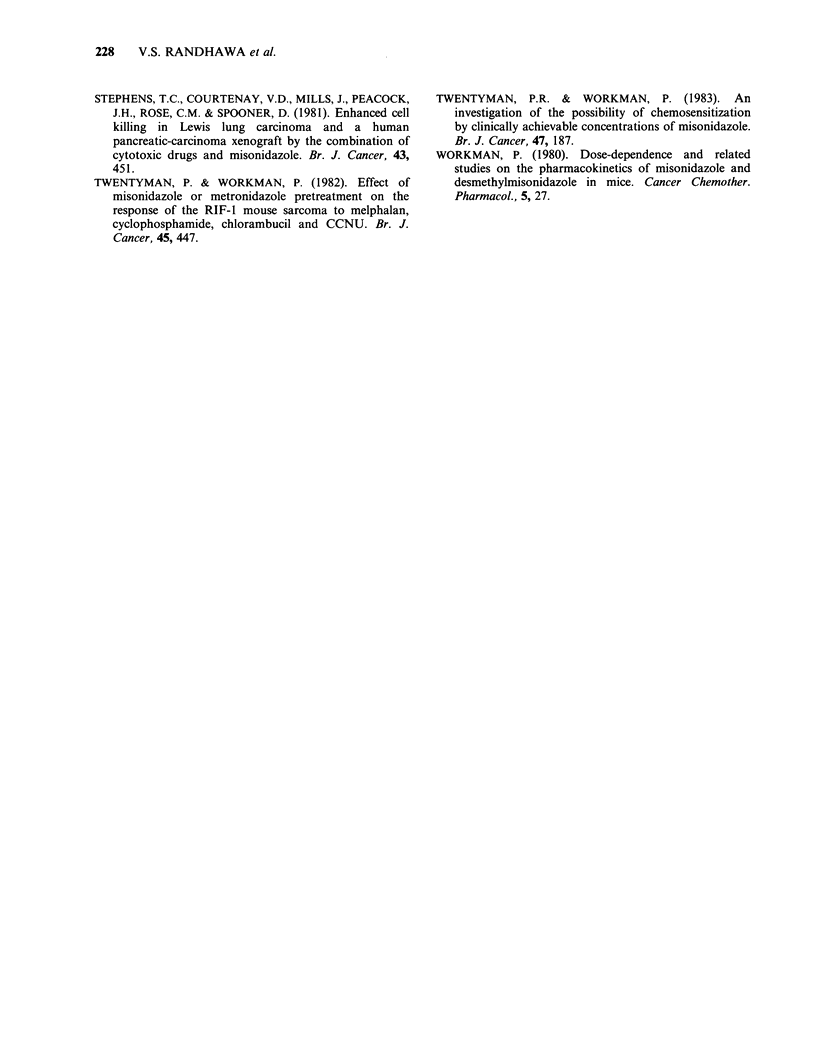# Factors influencing the chemosensitization of melphalan by misonidazole.

**DOI:** 10.1038/bjc.1985.32

**Published:** 1985-02

**Authors:** V. S. Randhawa, F. A. Stewart, J. Denekamp, M. R. Stratford

## Abstract

The effect of melphalan alone or combined with various schedules of misonidazole (MISO) has been tested on a murine fibrosarcoma. The tumoricidal effect has been determined using the growth delay assay. Large single doses (500-1000 mgkg-1) of MISO enhanced the anti-tumour effect of melphalan, especially at high melphalan doses. This was accompanied by a drop in body and tumour temperature and an increase in the melphalan half-life. The MISO-induced hypothermia was prevented in one experiment by keeping the mice in an ambient temperature of 35 degrees C for 3 h. This reduced the exposure to melphalan but did not diminish the cytotoxic effect of the drug combination. Chronic administration of MISO for an 8 h period gave no enhancement of melphalan damage, whether melphalan was given half-way through or at the end of the period of dosing. It seems that a threshold tumour concentration of MISO, in excess of 70 micrograms g-1, is needed for enhancement of melphalan cytotoxicity; prolonged exposures to very low doses are ineffective.


*Br. J. Cancer* (1985), **51**, 219–228

### Factors influencing the chemosensitization of melphalan by misonidazole

V.S. Randhawa, F.A. Stewart, J. Denekamp & M.R.L. Stratford

*Gray Laboratory of the Cancer Research Campaign, Mount Vernon Hospital, Northwood, Middlesex HA6 2RN, UK.*

**Summary** The effect of melphalan alone or combined with various schedules of misonidazole (MISO) has been tested on a murine fibrosarcoma. The tumoricidal effect has been determined using the growth delay assay. Large single doses (500–1000 mg kg$^{-1}$) of MISO enhanced the anti-tumour effect of melphalan, especially at high melphalan doses. This was accompanied by a drop in body and tumour temperature and an increase in the melphalan half-life. The MISO-induced hypothermia was prevented in one experiment by keeping the mice in an ambient temperature of 35°C for 3 h. This reduced the exposure to melphalan but did not diminish the cytotoxic effect of the drug combination.

Chronic administration of MISO for an 8 h period gave no enhancement of melphalan damage, whether melphalan was given half-way through or at the end of the period of dosing. It seems that a threshold tumour concentration of MISO, in excess of 70 $\mu$g g$^{-1}$, is needed for enhancement of melphalan cytotoxicity; prolonged exposures to very low doses are ineffective.

Hypoxic cell radiosensitizers such as misonidazole (MISO) have been shown to enhance the cytoxicity of certain chemotherapeutic agents in a variety of mouse tumours (see reviews by McNally, 1982; Millar, 1982; Siemann, 1982). The majority of the studies have been with single doses and most have shown that chemotherapeutic enhancement occurs after high doses of MISO which would, however, have no clinical relevance (McNally, 1982; Millar, 1982; Siemann, 1982). These single dose studies are difficult to translate directly into clinical terms, because of the 10-fold difference between mouse and man in the half-life of MISO, particularly since the cytotoxic effect to MISO is reported to be a supralinear function of exposure time (Hall *et al.*, 1978; Stratford & Adams, 1978). A few murine studies have been reported in which the human pharmacokinetics with lower plasma levels of MISO have been simulated by multiple injection schedules in order to assess the possible role of chemosensitization by MISO in the clinic (Hirst & Brown, 1982; McNally *et al.*, 1983; Twentyman & Workman, 1983). Some of these studies have demonstrated significant chemosensitization, but it is not a universal finding.

In this paper we report a comparison of the response of a fibrosarcoma (SA FA) to melphalan given in combination with large single doses of MISO, or small but prolonged exposures. The effect of each drug on the pharmacokinetics of the other has been measured, together with the influence of these two drugs on body and tumour temperature. In addition, the influence of MISO induced hypothermia has been investigated by maintaining some of the animals in a 35°C environment shortly after drug administration to prevent the hypothermia.

Correspondence: V.S. Randhawa.
Received 21 June 1984; and in revised form 25 October 1984.

### Materials and methods

### *Mice and tumours*

The fibrosarcoma SA FA grown in WHT/Gy f BSVS mice has been used. This tumour arose spontaneously and was maintained by serial passage in the inbred strain of origin at the Gray Laboratory for many years. For the last 4 years the cells have been kept in liquid nitrogen and no more than ten transplants have been used before taking fresh cells from the frozen store. All experiments have been performed with Category 4 specific pathogen free mice.

### *Drugs*

All drugs were freshly prepared on the day of the experiment. Melphalan was first dissolved in 0.5 ml of acid alcohol (2% HCl in ethanol) and then further diluted with 9.5 ml of sterile saline immediately before administration (final pH2). It was administered i.p. according to the body weight of each animal in a volume of 0.01 ml g$^{-1}$. In all the single dose studies MISO was administered i.p. immediately after the melphalan. For the chronic exposures 0.01 ml g$^{-1}$ of a standard MISO solution was used, to give a priming dose of 120 mg kg$^{-1}$,

© The Macmillan Press Ltd., 1985

followed by top up doses of $30 mg kg^{-1}$ every 20 min for 8 h.

### Assays

*Growth delay*

Tumours were implanted by trochar s.c. on the back and treated when they reached a mean diameter of $7.5 \pm 1$ mm. Each tumour was measured 2–3 times a week in 3 orthogonal diameters and the geometric mean diameter was calculated for each tumour. Dose response curves were constructed by assessing the time taken for each tumour to grow to 4.5 mm above the treatment size, thus obtaining a growth delay ($\pm$ s.e.) for each dose group (usually 6–10 animals). In those dose groups where the data have been pooled from several experiments, the number of mice in a group ranges from 19–54.

*Temperature measurements*

Rectal and tumour temperatures were taken on unanaesthetised mice using a special copper constantan thermocouple connected to a direct reading thermocouple amplifier (Bailey Instruments, USA). Mice were maintained in ambient temperatures of 21°C or 35°C.

*Pharmacology*

Blood was collected under penthrane anaesthesia from blood vessels in the neck and immediately cooled on ice in heparinized tubes. It was centrifuged at 4°C, the plasma removed and frozen in a liquid nitrogen/alcohol mixture at −70°C and stored at −20°C prior to analysis. Tumours were excised immediately after the blood collection and frozen in a similar manner. Blood and tumour concentrations of MISO were determined by high performance liquid chromatography (HPLC) using a method similar to that described by Dische *et al.* (1979). For melphalan analysis, aliquots of plasma were deproteinised with 4 vol of acetonitrile containing 1% HCl, mixed and centrifuged; an aliquot of supernatant was dried on a Savant sample concentrator. Tumours were homogenized in 3 vol of 10 mM HCl and an aliquot was deproteinised with acetonitrile, mixed, centrifuged and the supernatant taken to dryness. Samples were then suspended in eluent and injected on to the HPLC column. The conditions used were 40% acetonitrile, 5 mM heptane sulphonic acid, 2 mM dibutylamine, 40 mM orthophosphoric acid, 10 mM sodium dihydrogen orthophosphate, pH 2.7. A flow rate of $2 ml min^{-1}$ was used with a Waters Wisp injector, hypersil ODS column, Waters 441 UV detector operating at 254 nm and a Waters 730 data module.

### Results

The response of the tumour SA FA to graded doses of melphalan is plotted in Figure 1, as additional time taken to grow from 7.5 to 12 mm mean diameter relative to controls. Untreated tumours took 12–14 days to reach this size. In Panel A the response is shown to graded doses of melphalan given alone or shortly before MISO. This represents pooled data from 3–8 experiments and contains 19–54 mice per point. Increasing growth delay was seen with increasing melphalan dose. One thousand $mg kg^{-1}$ MISO (given without melphalan) gave ~2 days delay in growth. This MISO dose also enhanced the growth delay obtained with all melphalan doses, especially at the higher doses. A much smaller but significant, sensitization was also seen with $500 mg kg^{-1}$ MISO although no delay was seen with an intermediate dose ($670 mg kg^{-1}$) of MISO given alone (data not shown). The growth delays obtained by melphalan or MISO alone or when MISO was combined with $10 mg kg^{-1}$ of melphalan are given in Table I. Panel B in Figure 1 shows growth delay data obtained using chronic administration of MISO over an 8 h period, with graded doses of melphalan being administered either halfway through (i.e. at 4 h) or at the end of the chronic MISO administration. This more closely simulates what might occur in man where low MISO doses would be given, but the longer biological half-life in humans would maintain the MISO levels for many hours. No significant sensitization was seen with either of the chronic dosage regimes, at any dose level (Table I).

A similar experiment was performed with cyclophosphamide and the results were essentially the same (data not shown). A significant enhancement was seen with $1000 mg kg^{-1}$ MISO at all cyclophosphamide doses ranging from 40–$160 mg kg^{-1}$. However, no enhancement was seen at any dose level with an 8 h chronic MISO exposure, whether the cyclophosphamide was given in the middle or at the end of the chronic MISO schedule.

*Temperature effects*

Several authors have reported changes in body temperatures after high doses of MISO and these could contribute to the observed effects on the tumour. We have therefore measured the temperatures of both the tumour and the body core for a period of 5–6 h after drug administration. The results are shown in Table I. At 21°C the rectal temperatures of untreated mice were in the range of

**Figure 1** Growth delay as a function of melphalan dose (a) (○) melphalan alone (19–56 tumours/point); (●) melphalan + 1000 mg kg$^{-1}$ MISO (19–56 tumours/point). (b) (▼) chronic exposure to 70 μg g$^{-1}$ MISO for 8 h with melphalan given halfway through (8–10 tumours per point); (▲) chronic exposure to 70 μg g$^{-1}$ MISO for 8 h with melphalan given at the end (8–10 tumours per point); (○) melphalan alone (8 tumours per point). Enhancement is only seen with the large MISO dose.

**Table I** Effects of administering MISO or melphalan alone or in combination

| *Conditions* | | | *Temperature drop °C* | | | | *MISO* | | *Melphalan* | | *Tumour response* |
|---|---|---|---|---|---|---|---|---|---|---|---|
| *MISO mg kg$^{-1}$* | *Melphalan mg kg$^{-1}$* | *Ambient temp °C* | *½–3 h Rectal* | *½–3 h Tumour* | *3–6 h Rectal* | *3–6 h Tumour* | *Tumour Peak conc μg g$^{-1}$* | *Tumour AUC μg h$^{-1}$* | *Plasma $T_{½}$ min* | *Tumour AUC μg min$^{-1}$* | *Growth delay (days)* |
| *Acute* | | | | | | | | | | | |
| 1000 | — | 21 | ~5 | ~5 | ~4 | ~4 | 702±27 | 1545±195 | — | — | 2.3±0.6 |
| 1000 | 10 | 21 | ~5 | ~5 | ~4 | ~4 | NA | NA | 59±7 | 924±267 | 12.8±1.2 |
| 1000 | — | 35 | ~1 | ~1 | ~3 | ~3 | 603±135 | 1714±283 | — | — | 0 |
| 1000 | 10 | 35 | ~1 | ~1 | ~3 | ~3 | NA | NA | 48±13 | 558±172 | 17.9±7.6 |
| 500 | — | 21 | ~3 | ~3 | ~1 | ~0.5 | 223±19 | 402±78 | — | — | 0 |
| 500 | 10 | 21 | ~3 | NA | ~1 | NA | NA | NA | 41±6 | 626±150 | 6.1±1.1 |
| 0 | 10 | 21 | 0 | 0 | 0 | 0 | — | — | 24±3 | 316±69 | 3.1±0.7 |
| 0 | 10 | 35 | 0 | 0 | 0 | 0 | — | — | 24±4 | 364±101 | 2.6±1.0 |
| *Chronic* | | | | | | | | | | | |
| 8 h | — | 21 | ~1 | ~1 | ~1 | ~1 | ~70 | 573±60 | — | — | 0 |
| 8 h | 10(4) | 21 | NA | NA | NA | NA | NA | NA | NA | NA | 7.0±0.9 |
| 8 h | 10(8) | 21 | NA | NA | NA | NA | NA | NA | 30±6 | 275±67 | 6.5±0.7 |
| 0[a] | — | 21 | 0 | 0 | 0 | 0 | — | — | — | — | 0 |
| 0 | 10 | 21 | NA | NA | NA | NA | — | — | 26±2[a] | 344±89[a] | 5.3±0.7 |

NA—data not available.
[a]With chronic administration of saline every 20 min for 8 h.

38.0±0.5°C, but the tumour temperatures were always ~3°C lower (35.2±0.2°C). Large single doses of MISO caused a temperature drop which was dose dependent and which persisted for at least 5 h in animals kept at normal room temperature (21°C). This hypothermia was apparent in both rectal and tumour measurements. They both fell by 3°C after 500 mg kg$^{-1}$ and by ~5°C after 1000 mg kg$^{-1}$. In this way the initial difference in temperature between the tumour and the body core was maintained (Table I). The addition of 10 mg kg$^{-1}$ melphalan did not alter this hypothermic response.

Maintaining the mice in a warm room at 35°C for 3 h after administration of the drugs prevented the rectal temperature falling below 35.5°C, and the tumours were in the range 34–36°C. Even after 3 h, however, a prompt drop in body and tumour temperature resulted when the animals were returned to a 21°C environment. Again the presence of melphalan did not influence this MISO-induced hypothermia.

There was a decrease by ~1°C in the rectal and tumour temperatures of mice given repeated MISO injections compared with those given an equivalent volume of saline using the same schedule. These temperature variations were small compared to those after large single doses, and did not lead to consistent or prolonged hypothermia.

The influence of this hypothermic effect of MISO on the chemosensitivity was investigated in one experiment by keeping mice at different ambient temperatures for 3 h after drug administration. The results are shown in Figure 2. Mice from the same transplant were randomly allocated to the two temperature regimes. In the left hand panel the response of mice kept at room temperature is shown. The effect of MISO in this particular experiment is similar to that for the pooled data in Figure 1A. The area enclosed by ± s.e. on each point is reproduced (Figure 2B) for comparison with the response of tumours maintained for 3 h at 35°C after receiving the drugs. There was no significant differences in the effect of melphalan alone but the response of mice treated with both drugs at 35°C was consistently greater than those maintained at room temperature for all melphalan doses above 2.5 mg kg$^{-1}$. However, this treatment was also more toxic; 3/8 and 5/8 mice died within 7 days after 7 mg kg$^{-1}$ and 10 mg kg$^{-1}$ melphalan when it was combined with MISO and the elevated temperature, compared with no deaths at room temperature. A further experiment was performed in which the melphalan dose was kept constant at

Figure 2 Effect of ambient temperature on the tumour response to melphalan alone or combined with 1000 mg kg$^{-1}$ MISO. (a) 21°C; (b) 35°C. The enhancement by MISO persists when the mice are kept at 35°C for 3 h after drug administration. Hatched area in panel (b) is represented from panel (a).

10 mg kg$^{-1}$ and the MISO dose was varied from 200 to 1000 mg kg$^{-1}$ (data not shown). As in Figure 2B an increased effect of the drug combination was seen if the mice were kept at 35°C instead of at 21°C.

*Pharmacokinetics*

The effect of drug dose and body temperature on the pharmacokinetics of both drugs has been studied. Melphalan (10 mg kg$^{-1}$) has no effect on the pharmacokinetics of MISO (1000 mg kg$^{-1}$) at 21°C or 35°C (data not shown). Figure 3 shows the concentration of MISO (in the absence of melphalan) in blood and tumour as a function of time after injection. Each point represents the mean of 3 mice ± s.e. The top panels show the data after a large single dose for mice kept at 21°C (A) and at 35°C for 8 h after drug administration (B). For both groups the tumour concentration stayed consistently below that in blood for at least 6 h. The peak tumour level was reached more rapidly in mice at 35°C than in those at room temperature, and the clearance of the MISO from both blood and tumour was slower. Panel C shows that lower blood and tumour levels were achieved after 500 mg kg$^{-1}$ MISO, approximately in proportion to the administered dose. During the chronic MISO administration (panel D) a fairly constant level of ~100 μg ml$^{-1}$ was achieved in blood, and 70 μg g$^{-1}$ in the tumour.

The peak concentration and the exposure dose, calculated from the area under each curve (AUC) for tumour MISO levels are shown in Table I. Although an 8 h exposure in the warm room was used for the pharmacology this was subsequently found to be too toxic for the growth delay experiments; animals died within a week after

**Figure 3** Misonidazole concentrations in blood (●) and tumour (□) after the four schedules. The tumour concentration generally stays below that in blood. Each point is the mean ± s.e. for 3 animals. (a) 1000 mg kg$^{-1}$ MISO acute 21°C; (b) 1000 mg kg$^{-1}$ MISO acute 35°C; (c) 500 mg kg$^{-1}$ MISO acute 21°C; (d) chronic MISO 21°C.

**Figure 4** Melphalan concentrations in tumours after 10 mg kg$^{-1}$ melphalan alone (○) or combined with MISO (●). Large single doses of MISO prolong the melphalan half life (a–c) but chronic low doses have no effect (d). Each point represents the mean ± s.e. of 3 animals.

giving both drugs and maintaining them for 8 h at 35°C. The AUC for MISO for mice maintained at 35°C in Table I may therefore be a slight overestimate. However, since the concentration had fallen to 10% of peak levels by 3 h this is unlikely to be a major source of error.

The melphalan concentration was determined for both tumour and plasma in mice given melphalan (10 mg kg$^{-1}$) or combined with MISO. The plasma melphalan half lives are shown in Table I. The tumour results are shown in Figure 4 for all the doseage regimes. The single doses of MISO prolonged the melphalan half life, but there was no influence on melphalan pharmacokinetics when low levels of MISO were maintained for 8 h. The tumour exposure to melphalan (i.e. area under the curve) is listed in Table I.

### Discussion

These data demonstrate that the chemosensitizing effects of MISO which can be shown with large doses are lost if, in an attempt to mimic the likely pharmacokinetics in human tumours, the drug is given as a low-dose chronic exposure. No enhancement of melphalan (or cyclophosphamide) cytotoxicity was observed when it was given either halfway through or at the end of an 8 h chronic MISO administration. This is, of course, disappointing for the clinical application of MISO in chemotherapy.

The other published studies of MISO and melphalan combinations are summarised in Table II, and a similar conclusion can be drawn from them. With large single doses a significant effect is

**Table II** Efficacy of melphalan combined with MISO: Comparison of literature reports.

*Single doses*

| *Author* | *Tumour* | *MISO dose* $mg\,kg^{-1}$ | *Enhancement Ratio* |
|---|---|---|---|
| Clement *et al.* (1980) | M5076 | 600–1000 | 1.9–2.2 |
| Fu *et al.* (1981) | SQ 1 | 500 | No effect |
| Martin *et al.* (1981) | WH FIB | 1000 | 2.7[a] |
| McNally *et al.* (1983) | WH FIB | 800 | 2.7[a] |
| | WH FIB | 800 | 1.4–2.0 |
| Randhawa *et al.* (1982) | CA NT | 800 | 1.1–1.5 |
| | SA FA | 1000 | 1.8–5.3 |
| Rose *et al.* (1980) | Lewis Lung Ca. | 1000 | 2.0–2.7[a] |
| | | 330 | >1 |
| Sheldon *et al.* (1982) | MT | 500 | 1.7[a] |
| Stephens *et al.* (1981) | Lewis Lung Ca. | 750 | 2.0[a] |
| | H x 32 | 1000 | 1.9[a] |
| Twentyman & Workman (1982) | RIF I | 500 | No effect |
| | RIF I | 500 | No effect[a] |
| | KHT | 500 | No effect |
| | EMT 6 | 500 | No effect |
| Clutterbuck *et al.* (1982) | HX 34 | 1000 | >1.0 |
| | HX 47 | 1000 | >1.0 |
| *Present Study* | SA FA | 1000 | $2.0 \pm 0.2$[b] |
| | SA FA | 1000 (35°C) | $4.3 \pm 1.0$[b] |
| | SA FA | 500 | $1.4 \pm 0.2$[b] |

*Chronic Administration*

| *Author* | *Tumour* | *MISO conc. in blood* $\mu g\,ml^{-1}$ | *MISO conc. in tumour* $\mu g\,g^{-1}$ | *Exposure time (h)* | *Enhancement ratio* |
|---|---|---|---|---|---|
| Hirst (1982) | RIF 1 | 100–200 | — | 7 | 2.0 |
| McNally *et al.* (1983) | WH FIB | 100 | — | 8 | No effect |
| | WH FIB | 100 | — | 8 | 1.8[a] |
| Twentyman & Workman (1983) | RIF I | 100 | — | 7 | No effect |
| *Present Study* | SA FA[c] | 100 | ~70 | 8 | No effect |
| | SA FA[d] | 100 | ~70 | 8 | No effect |

[a]Tumour response assessed by plating cells after excision. All other studies have used growth delay to assay the response.

[b]Enhancement ratio $= \dfrac{\text{MTD Melphalan alone } (15\,mg\,kg^{-1})}{\text{Melphalan dose with MISO}}$ to achieve the same growth delay.

[c]Melphalan given at the end of 8 h chronic administration.

[d]Melphalan given halfway through the 8 h chronic administration.

seen in every study: A threshold dose of 300–500 mg kg$^{-1}$ seems to be needed. Only two studies at doses of 500 mg kg$^{-1}$ or below have demonstrated chemosensitization (Rose *et al.*, 1980; Sheldon *et al.*, 1982) and two other studies have shown *no* effect at 500 mg kg$^{-1}$ (Fu *et al.*, 1981; Twentyman & Workman, 1982). Far fewer studies have been published using chronic MISO with melphalan. In these, two different tumours have been used, and in each case one study shows an extra cytotoxicity with the combination whilst the other shows none. With the RIF-1 tumour the same assay was used in different laboratories, whereas with WH FIB two different assays used by the same workers led to opposite conclusions. These studies were all performed with MISO levels of

~100 $\mu g\,ml^{-1}$ in blood, which are similar to those in the present study and also to those likely to be achieved in the clinic. The evidence for an increased effectiveness of melphalan combined with prolonged low dose MISO is seen from Tables I and II to be less than compelling.

As the MISO dose is increased above 200 $mg\,kg^{-1}$ the total exposure of tumour cells to the drug would change, partly because of the higher peak concentration and also due to the extension of the half-life seen at high MISO doses (Workman, 1980). Several *in vitro* studies have indicated that hypoxic cytotoxicity is more dependent upon exposure time (T) than peak concentration (C) (Hall *et al.*, 1978; Stratford & Adams, 1978) and has been correlated with a time squared expression i.e. cytotoxicity $\alpha C \times T^2$ (Hall *et al.*, 1978).

The present set of data allows us to analyse this for the four schedules which have been compared. In Figure 5 the additional delay resulting from MISO treatment (relative to give 10 $mg\,kg^{-1}$ melphalan alone) has been plotted as a function of the tumour exposure to MISO, calculated from $C \times T$ (panel A) or form $C \times T^2$ (panel B). The data for chronic exposures fall below the data for acute single doses in both panels but the discrepancy is even greater for the $C \times T^2$ calculation than for the simpler $C \times T$. If MISO cytotoxicity plays a role in chemosensitization then these *in vivo* results do not support the concept of cytotoxicity being a supralinear function of overall time. Rather they support the view that a critical threshold level is needed, regardless of exposure time, to achieve additional tumour cell kill.

Large MISO doses cause a drop in body and tumour temperature which may influence many physiological and biochemical parameters, including respiration and heart rate, blood flow to tumour and normal tissues and the rate of various biochemical processes. The hydrolysis of melphalan, and its interaction with DNA (alkylation), could also be influenced by the changes in temperature. Figure 4 and Table I demonstrate that changes in the rate of melphalan removal from the plasma and tumour occur with large single doses of MISO. The extended plasma half-life has been reported by Stephens *et al.* (1981), Clutterbuck (1982) and Hinchliffe *et al.* (1983), but tumour measurements have not previously been available. Since changes in melphalan pharmacokinetics occur with high MISO doses it is possible that the chemosensitization observed with melphalan and MISO can be explained by increased exposure of the tumour cells to melphalan.

In Figure 5C, the additional delay from the combined treatment has been plotted as a function of tumour exposure to melphalan (calculated as $C \times T$). For the 21°C data alone, it could be argued that the changes in melphalan exposure to the tumour could explain the observed effect. However, when the mice are maintained at 35°C the extension of the melphalan $T_{1/2}$ and hence the exposure dose are both reduced, yet the combined treatment is more effective than any of the others, whereas the effect of melphalan alone is unchanged (Figure 2B). It is likely that several competing processes are occurring and the overall balance between them under different conditions will determine the

**Figure 5** MISO induced delay when combined with 10 $mg\,kg^{-1}$ melphalan plotted as a function of MISO or melphalan exposure dose. (a) MISO dose = AUC; (b) MISO dose = concentration × time$^2$; (c) melphalan dose = AUC. (■) 500 $mg\,kg^{-1}$; (◆) 1000 $mg\,kg^{-1}$, 21°C; (●) 1000 $mg\,kg^{-1}$, 35°C; (▼) chronic MISO, with melphalan after 4 h; (▲) chronic MISO, with melphalan after 8 h.

response observed. Enhanced alkylation and/or reduced repair of sublethal lesions in the DNA may both be involved.

The effects with cyclophosphamide were very similar i.e. an increase in tumour growth delay after large single doses of MISO. All the factors mentioned above would apply to this result, but in addition the rate at which the cyclophosphamide is metabolised to its active form could also be influenced by changes in body temperature.

Sieman (1984) recently reviewed the field of electron affinic radiosensitizers when combined with a variety of chemotherapeutic agents. He concluded that no single unifying mechanisms for chemosensitization exists and that changes in drug pharmacokinetics, cellular SH levels and repair of DNA damage are all involved. He also concluded that, although a therapeutic gain has generally been seen for large sensitizer doses, the results with clinically achievable dose levels needed further evaluation.

The present results indicate that low dose chronic MISO administration is ineffective, in this fibrosarcoma when combined with melphalan or cyclophosphamide. The chemosensitization by large single doses does not appear to be an artefact of the hypothermia that accompanies it, but rather seems to indicate that a critical peak MISO concentration ($>70\,\mu g\,g^{-1}$) must be achieved in the tumour (Figure 3 and Table I). This could be attained clinically with large infrequent MISO doses, but the number of doses would be limited by the cumulative toxicity. However, since chemotherapy is usually given as large infrequent doses over many months, the toxicity experience from repeated smallish dosing in radiotherapy studies 2–5 times a week over 6–8 weeks may be a pessimistic guide to the tolerable MISO dose if it were combined with chemotherapy.

We are grateful to Roche Products Ltd., Welwyn Garden City, Herts, for misonidazole; Wellcome Foundation Ltd., Crewe, Cheshire, for melphalan; Boehringer Ingelheim Ltd., Bracknell, Berks, for cyclophosphamide and the Cancer Research Campaign for financing this work. We should like to thank Mr P. Russell and the animal house staff for care of the mice, Mrs J. Wilson and Mrs J. Arnold for secretarial assistance and Prof. J.F. Fowler and Dr S.A. Hill for constructively critical comments.

### References

CLEMENT, J.J., GORMAN, M.S., WODINSKY, I., CATANE, R. & JOHNSON, R.K. (1980). Enhancement of antitumor activity of alkylating agents by the radiation sensitizer misonidazole. *Cancer Res.*, **40**, 4165.

CLUTTERBUCK, R.D., MILLAR, J.L. & McELWAIN, T.J. (1982). Misonidazole enhancement of the action of BCNU and melphalan against human melanoma xenografts. *Am. J. Clin. Oncol.*, **5**, 73.

DISCHE, S., SAUNDERS, M.I., FLOCKHART, R., LEE, M.E. & ANDERSON, P. (1979). Misonidazole – a drug for trial in radiotherapy and oncology. *Int. J. Radiat. Oncol. Biol. Phys.*, **5**, 851.

FU, K., PHILLIPS, T.L., RAYNER, P.A. & ROSS, G.Y. (1981). The combined effects of misonidazole and chemotherapy on tumour and normal tissue *in vivo*. Abstract. *Proceedings of the 29th Annual Meeting of the Rad. Res. Soc.*, p. 90.

HALL, E.J., MILLER, R., ASTOR, M. & RINI, F. (1978). The nitroimidazoles as radiosensitizers and cytotoxic agents. *Br. J. Cancer*, **37**, (Suppl. III), 120.

HIRST, D.G. & BROWN, J.M. (1982). The therapeutic potential of misonidazole enhancement of alkylating agent cytotoxicity. *Int. J. Radiat. Oncol. Biol. Phys.*, **8**, 639.

HINCHLIFFE, M., MCNALLY, N.J. & STRATFORD, M.R.L. (1983). The effect of radiosensitizers on the pharmacokinetics of melphalan and cyclophosphamide in the mouse. *Br. J. Cancer*, **48**, 375.

MARTIN, W.M.C., McNALLY, N.J. & De RONDE, J. (1981). Enhancement of the effect of cytotoxic drugs by radiosensitizers. *Br. J. Cancer*, **43**, 756.

MILLAR, B.C. (1982). Hypoxic cell radiosensitizers as potential adjuvants to conventional chemotherapy for the treatment of cancer. *Biochem. Pharmacol.*, **31**, 2439.

McNALLY, N.J., HINCHLIFFE, M. & De RONDE, J. (1983). Enhancement of the action of alkylating agents by single high, or chronic low doses of misonidazole. *Br. J. Cancer*, **48**, 271.

RANDHAWA, V.S., STEWART, F.A. & DENEKAMP, J. (1982). Chemosensitization of mouse tumours by misonidazole. *Int. J. Radiat. Oncol. Biol. Phys.*, **8**, 671.

ROSE, C.M., MILLAR, J.L., PEACOCK, J.H., PHELPS, T.A. & STEPHENS, T.C. (1980). Differential enhancement of melphalan cytotoxicity in tumor and normal tissue by misonidazole. In: *Radiation Sensitizers Their Use in the Clinical Management of Cancer*. (Ed. Brady), USA: Masson Publ., p. 250.

SHELDON, P.W., BATTEN, E.L. & ADAMS, G.E. (1982). Potentiation of melphalan activity against a murine tumour by nitroimidazole compounds. *Br. J. Cancer*, **46**, 525.

SIEMANN, D.W. (1982). Potentiation of chemotherapy by hypoxic cell radiation sensitizers – A review. *Int. J. Radiat. Oncol. Biol. Phys.*, **8**, 1029.

SIEMANN, D.W. (1984). Modification of chemotherapy by nitroimidazoles. *Int. J. Radiat. Oncol. Biol. Phys.*, **10**, (In press).

STRATFORD, I.J. & ADAMS, G.E. (1978). The toxicity of the radiosensitizer misonidazole towards hypoxic cells *in vitro*: A model for mouse and man. *Br. J. Radiol.*, **51**, 745.

STEPHENS, T.C., COURTENAY, V.D., MILLS, J., PEACOCK, J.H., ROSE, C.M. & SPOONER, D. (1981). Enhanced cell killing in Lewis lung carcinoma and a human pancreatic-carcinoma xenograft by the combination of cytotoxic drugs and misonidazole. *Br. J. Cancer*, **43**, 451.

TWENTYMAN, P. & WORKMAN, P. (1982). Effect of misonidazole or metronidazole pretreatment on the response of the RIF-1 mouse sarcoma to melphalan, cyclophosphamide, chlorambucil and CCNU. *Br. J. Cancer*, **45**, 447.

TWENTYMAN, P.R. & WORKMAN, P. (1983). An investigation of the possibility of chemosensitization by clinically achievable concentrations of misonidazole. *Br. J. Cancer*, **47**, 187.

WORKMAN, P. (1980). Dose-dependence and related studies on the pharmacokinetics of misonidazole and desmethylmisonidazole in mice. *Cancer Chemother. Pharmacol.*, **5**, 27.